# Detection of Ultrasonic Stress Waves in Structures Using 3D Shaped Optic Fiber Based on a Mach–Zehnder Interferometer

**DOI:** 10.3390/s18041218

**Published:** 2018-04-16

**Authors:** Chengming Lan, Wensong Zhou, Yawen Xie

**Affiliations:** 1 School of Civil and Resource Engineering, University of Science & Technology Beijing, Beijing 100083, China; lanchengming@ustb.edu.cn; 2 Key Lab of Structures Dynamic Behavior and Control of the Ministry of Education, Harbin Institute of Technology, Harbin 150090, China; 13104627301@163.com; 3 Key Lab of Smart Prevention and Mitigation of Civil Engineering Disasters of the Ministry of Industry and Information Technology, Harbin Institute of Technology, Harbin 150090, China; 4 School of Civil Engineering, Harbin Institute of Technology, Harbin 150090, China

**Keywords:** ultrasonic wave, acoustic emission, optic fiber, Mach–Zehnder interferometer

## Abstract

This work proposes a 3D shaped optic fiber sensor for ultrasonic stress waves detection based on the principle of a Mach–Zehnder interferometer. This sensor can be used to receive acoustic emission signals in the passive damage detection methods and other types of ultrasonic signals propagating in the active damage detection methods, such as guided wave-based methods. The sensitivity of an ultrasonic fiber sensor based on the Mach–Zehnder interferometer mainly depends on the length of the sensing optical fiber; therefore, the proposed sensor achieves the maximum possible sensitivity by wrapping an optical fiber on a hollow cylinder with a base. The deformation of the optical fiber is produced by the displacement field of guided waves in the hollow cylinder. The sensor was first analyzed using the finite element method, which demonstrated its basic sensing capacity, and the simulation signals have the same characteristics in the frequency domain as the excitation signal. Subsequently, the primary investigations were conducted via a series of experiments. The sensor was used to detect guided wave signals excited by a piezoelectric wafer in an aluminum plate, and subsequently it was tested on a reinforced concrete beam, which produced acoustic emission signals via impact loading and crack extension when it was loaded to failure. The signals obtained from a piezoelectric acoustic emission sensor were used for comparison, and the results indicated that the proposed 3D fiber optic sensor can detect ultrasonic signals in the specific frequency response range.

## 1. Introduction

Ultrasonic stress waves, either passive acoustic emission signals or active guided waves, propagating in a structure can be employed to interpret the structural status, especially to detect small structural damage, such as debonding, lamination, crack, corrosion, etc. Over the past few decades, the corresponding methods and techniques have also been proven effective and promising in a variety of metal, composite, concrete and other materials and structures [1,2,3,4,5,6,7,8]. Ultrasonic stress waves, generated by either transducers or a rapid release of energy within a stressed material, are often at a fairly high frequency, from several tens of kHz to approximately several MHz. These waves are captured usually by ultrasonic transducers, including piezoelectric wafers [7,9,10], magnetostrictive sensors [11,12], electromagnetic acoustic transducers (EMAT) [13], macro-fiber composites [14,15], etc. The aforementioned ultrasonic transducers are used to convert mechanical energy into electric or magnetic energy; therefore, they are more easily affected by electromagnetic interference. Moreover, they also suffer from distinct disadvantages, such as a larger footprint, and a lack of capability for continuous or long-term performance monitoring, such as corrosion-resistant and electrical stability. Recently, the developments in fiber optic sensors (FOSs) have provided an excellent substitution because optical fiber sensors have very small dimensions and are lightweight; they can be embedded unobtrusively within structures; they have a wide range of operating temperatures and are capable of transmitting a signal over a long distance; significantly, they resist the corrosion and are immune to electromagnetic interference [16].

A variety of optical sensing technologies have been developed over the years [17,18,19,20,21,22], and conventional FOSs are generally used to measure single-point or distributed strain [23] and temperature in the low frequency range, which is often lower than kHz. However, by using phase demodulation methods, FOSs can detect an ultrasonic signal with fairly high frequency, which is from several kHz to several MHz. This frequency range is usually used by ultrasonic guided waves based or acoustic emission methods for damage detection in civil, mechanical or aerospace structures. In 1977, Bucaro et al. [24] of the U.S. Naval Research Laboratory demonstrated the possibility of using an optical fiber to sense an acoustic field, which was produced by exciting a piezoelectric plate with a sinusoidal signal in a water-filled tank. Experiments were carried out over the 40–400 kHz frequency range. Gachagan et al. [25,26] proposed a condition monitoring system that used a fiber-optic sensor. Two groups of optical fibers were embedded across the composite plate, and a 633 nm Mach–Zehnder interferometer was used to demodulate the acoustic signal, which was a Lamb wave propagating in the plate. Using this system, a delamination through the thickness of the composite plate was localized by analyzing reflected Lamb waves successfully in the laboratory. These experimental results can be considered as a fundamental basis for structure health monitoring using the Mach–Zehnder interferometer-based ultrasonic FOSs (UFOSs). The fiber-optic sensor based on Mach–Zehnder can be also the distributed one [27], which has been used to detect leakage in the long pipeline. In addition, using a Mach–Zehnder interferometer on different structures, other demodulation techniques are also employed for detecting ultrasonic elastic signals [28], such as Michelson interferometers [29,30], Fabry–Perot interferometers [31,32], Sagnac interferometers [33], and Fiber Bragg Grating [34], or methods based on the Doppler effect of light wave transmission in an optical fiber [35,36]. It should be noted that a considerable part of the sensors above were used to detect acoustic emission signals.

In the aforementioned modulation techniques, the sensitivity of the UFOSs is closely related to the effective length of the UFOS; the larger the *L*, the larger the sensitivity. In early experiments, an optical fiber was attached to the structural surface or embedded within the structure along the whole dimension as a line. This layout first leads to the directionality of UFOSs; second, it cannot be used to estimate the source location even if two optical fibers were used, since they measure a line rather the the single point. Another method is the use of the circular loop UFOS, which can obtain higher sensitivity with a smaller area [36,37]. Its sensitivity can be controlled by changing the radius and number of turns, and the circular UFOS is not directional. However, in order to achieve further improved sensitivity, the circular UFOS will require a significantly large footprint. Additionally, in engineering practices, especially for the civil engineering structures, the sensor should be attached to the structural surface, which is often not well-prepared for reliable measurement. Recently, a fiber-optic ring sensor was presented by Wei et al. [38]. The optical fiber was around the acrylic cylindrical skeleton. It can be analyzed that the resonant frequency of the sensor is related to the resonant frequency of the skeleton, which is high for the solid one. In addition, this work was demonstrated on only a small aluminum plate for acoustic emission detection.

In this work, a 3D shaped optic fiber sensor, which is a hollow metal cylinder wrapped in optical fibers, is proposed to detect the ultrasonic stress waves. The metal hollow cylinder will be attached to the testing surface using different couplants, such as grease or superglue. In this case, removing the sensor from the structural surface will not damage the optical fiber. Moreover, this sensor may be designed by changing the geometric parameters and material, and provides the lesser and designable resonant frequency range.

In the remaining part of this paper, the basic working principle of a Mach–Zehnder interferometer will be presented first. Subsequently, a finite element analysis with thin aluminum plates using the proposed sensor is performed in detail to examine the feasibility of ultrasonic signal measurement. Finally, in order to explore the characteristics of the proposed sensor further, it is applied on the aluminum and reinforced concrete beam, respectively. The setups and results of the experimental test program will be presented and discussed. The damage progress in a concrete beam was monitored during a four-point bending test and acoustic emissions were detected using the proposed sensor and a piezoelectric (PZT) wafer for comparison.

## 2. Mach–Zehnder Interferometer and the Fiber Optic Sensor

### 2.1. The Principle of Mach–Zehnder Interferometers

**Figure 1:**
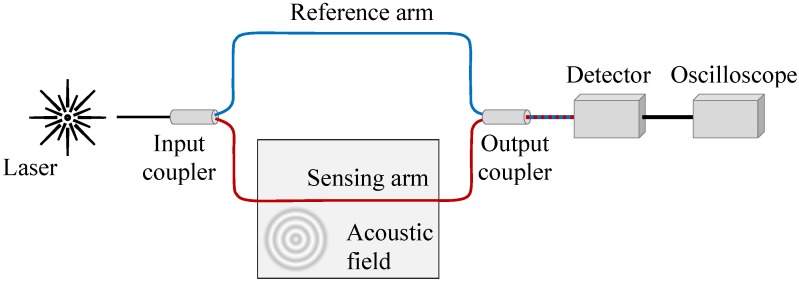
Schematic of a Mach–Zehnder interferometer.

The pressure of the ultrasonic stress signal may induce optical phase modulation within the optical fiber sensor bonded or embedded in the structures. The phase change can be demodulated using a Mach–Zehnder interferometer, whose principle is presented schematically in Figure 1. It is used to determine the relative phase shift variations between two light beams in two optical fibers, which are derived by splitting light from the same light source and, thus, they have exactly the same initial phase. In case the light propagating in one optical fiber is disturbed by the ultrasonic stress signal, the phase change can be converted by the photodetector and then collected using a digital oscilloscope. It should be noted that a polarization controller and variable optical attenuator are not included on the reference arm, which would be useful to maximize a detector’s sensitivity, since this is a demonstration only.

The strain induced by the ultrasonic stress waves will lead to an optical phase shift in the sensing arm. In general, the relative phase change can be expressed using three strain components [39]:

(1) $$\Delta \phi \approx \beta L\varepsilon_{11}-\frac{1}{2}\beta Ln^{2}\left(p_{11}\varepsilon_{11}+p_{12}\varepsilon_{22}+p_{12}\varepsilon_{33}\right),$$

where β is the propagation constant of a single mode, $\beta =k_{0}n$, $k_{0}$ is a free-space propagation constant, and *n* is the core index of the optical fiber. Furthermore, *L* is the length of the optical fiber; $\varepsilon_{11}$ is the strain in the direction of light propagation, i.e., the longitudinal direction of the optical fiber; $\varepsilon_{22}$ and $\varepsilon_{33}$ are the transverse strain, i.e., the radial direction of the optical fiber; $p_{11}$, $p_{12}$, and $p_{13}$ are the elements of the strain-optic tensor for a homogeneous isotropic material. When the sensing arm is bonded onto the surface of the structure by an adhesive, the sensitivity of the phase change to these strains is expressed as [37]:

(2) $$\frac{\Delta \phi }{\varepsilon_{11}}\approx \beta L-\frac{1}{2}\beta Ln^{2}\left(p_{11}+2vp_{12}\right),$$

where *v* is the Poisson’s ratio of the optical fiber.

### 2.2. The 3D Shaped Ultrasonic Fiber Optic Sensor

**Figure 2:**
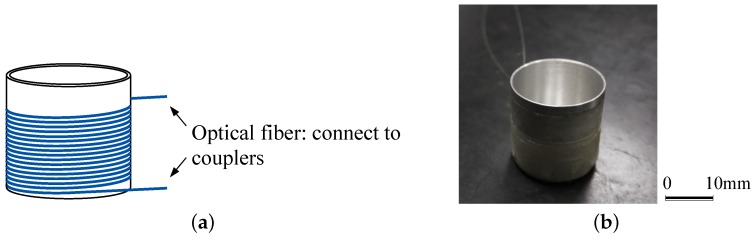
The 3D shaped optical fiber ultrasonic sensor: (**a**) sketch; (**b**) photo.

The aforementioned equations demonstrate that the sensitivity of the UFOSs based on a Mach–Zehnder interferometer is highly dependent on the length of the optical fiber. In this work, to obtain as large a response from the sensor as possible, the optical fiber is wrapped around a hollow cylinder with a base, as shown in Figure 2. With this shape, the sensor sensitivity increases, while its coverage is kept constant.

**Figure 3:**
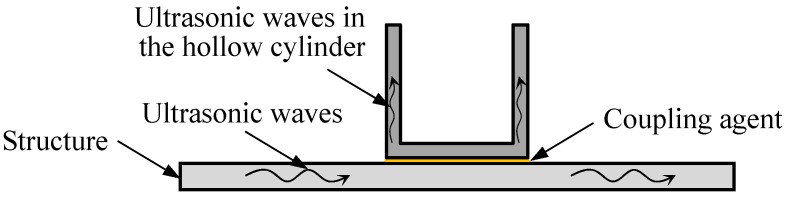
Propagating of ultrasonic waves from the structure to the sensor.

In this sensor, the optical fiber is not exposed to the structure directly, but receives the ultrasonic signals through the coupling between the hollow cylinder and the object structure, as indicated in Figure 3, which shows the guided wave case. Actually, in the object structure, ultrasonic stress waves can be not only guided waves but also bulk waves, surface waves , etc. In the interface between the hollow cylinder and the object structure, ultrasonic stress waves can be considered as asymmetric displacements applied on the hollow cylinder, even if they are the symmetric mode in the plate. Along the axial direction of the hollow cylinder, there are three guided wave modes, i.e. longitudinal mode (*L* mode), torsional mode (*T* mode), and flexural mode (*F* mode) [40]. The first two modes can be excited by the symmetric loading. In this work, guided waves propagating in the hollow cylinder will be *F* mode, since the excitation is asymmetric. The hollow cylinder is very short, and then the *F* mode guided waves reflect repeatedly. The displacement field on the surface of the hollow cylinder complies with the analytic wave equations of guided wave along the axial direction. Similarly, the deformation of the coiled optical fiber is produced due to the coupling between the fiber and the surface of the hollow cylinder. According to the theory of ultrasonic guided waves in the hollow cylinder, the displacement components related to the deformation of the optical fiber are the radial and circumferential ones, $u_{r}$ and $u_{\theta }$, which can be obtained analytically for the single guided wave mode. The length change for single loop $\Delta L_{i}$ can be obtained through the curvilinear integral. Thus, the total relative phase change of the optical fiber with *m* loops is

(3) $$\Delta \phi =\left[1-\frac{1}{2}n^{2}\left(p_{11}+2vp_{12}\right)\right]\beta \sum_{i=1}^{m}\Delta L_{i}.$$

However, in case of multiple wave reflections, this problem is difficult to analyze theoretically. However, it can still be concluded that the energy into the hollow cylinder is dependent on the diameter of the hollow cylinder and the wavelength. A smaller stiffness of the cylinder wall results in a lower resonant frequency and a larger response; therefore, the response of the optical fiber is also dependent on the stiffness of the cylinder wall. Notably, when the optical fiber bends too much, there will be a large optical power less; therefore, a small diameter of the hollow cylinder is avoided. An appropriate diameter is used in this work.

In addition, in regard to the signal characteristics in the frequency domain, the ultrasonic signals are reflected repeatedly within the hollow cylinder, which results in complicated overlapping signals; however, it is fairly easy to demonstrate that the main frequency characteristic is not affected significantly, as shown in Equations (4) and (5):

(4) $$F\left(\omega \right)=\int_{-\infty }^{+\infty }f\left(t\right)e^{-j\omega t}dt,$$

where f(t) is a narrow-band signal, which is used widely in guided ultrasonic wave applications. F(ω) is the amplitude in the frequency domain:

(5) $$\begin{array}{cc} F^{\prime }\left(\omega \right) & =\int_{-\infty }^{+\infty }\left[a_{1}f\left(t-t_{1}\right)+a_{2}f\left(t-t_{2}\right)+\ldots +a_{n}f\left(t-t_{n}\right)\right]e^{-j\omega t}dt\\ & =\left[a_{1}e^{j\omega t_{1}}+a_{2}e^{j\omega t_{2}}+\ldots ..+a_{n}e^{j\omega t_{n}}\right]F\left(\omega \right), \end{array}$$

where $a_{i}$ is the scale factor of the amplitude, and $t_{i}$ is the time delay for each ultrasonic signal. The sum in the above equation indicates the superposition of all signals, which have different amplitudes and different arrival time, in the time domain. Equation (5) gives the amplitude in the frequency domain of the overlapping signal. It shows that $F^{\prime }\left(\omega \right)$ has a similar predominant frequency to F(ω).

## 3. Feasibility Analysis Using the Finite Element Model

**Figure 4:**
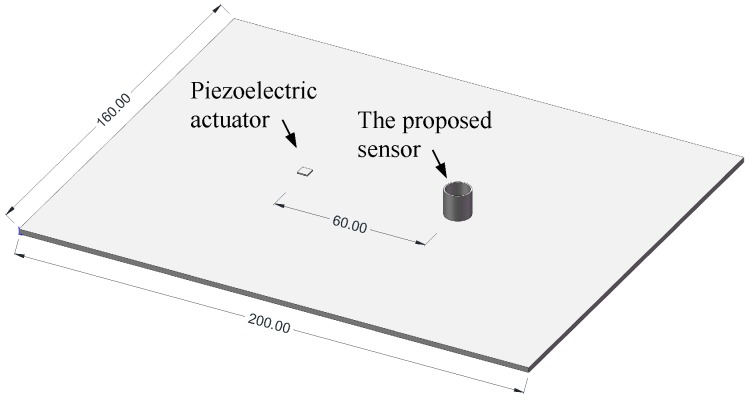
Layout of the piezoelectric actuator and the proposed sensor in the finite element model.

Finite element analyses were first conducted on a metallic plate to explore the basic sensing ability of the proposed sensor. A plate (200 mm × 160 mm × 1.6 mm) was modeled as aluminum material using a SOLID185 element with ANSYS. Two square-shaped piezoelectric wafers (4 mm × 4 mm), modeled using a SOLID5 element, were bonded onto both the upper and lower surfaces and the exact same position, working as actuators, which can generate a single mode Lamb wave propagating in the plate by applying symmetric or anti-symmetric electric fields. An aluminum hollow cylinder with a base, an outer radius of 5 mm, and an inner radius of 4.6 mm represents the proposed sensor, which was only used to demonstrate the wave scattering; therefore, there is no optical fiber surrounding it. The proposed sensor was placed 60 mm away from the actuator. Figure 4 shows the size of the plate and the layout of the piezoelectric actuator and the proposed sensor.

**Figure 5:**
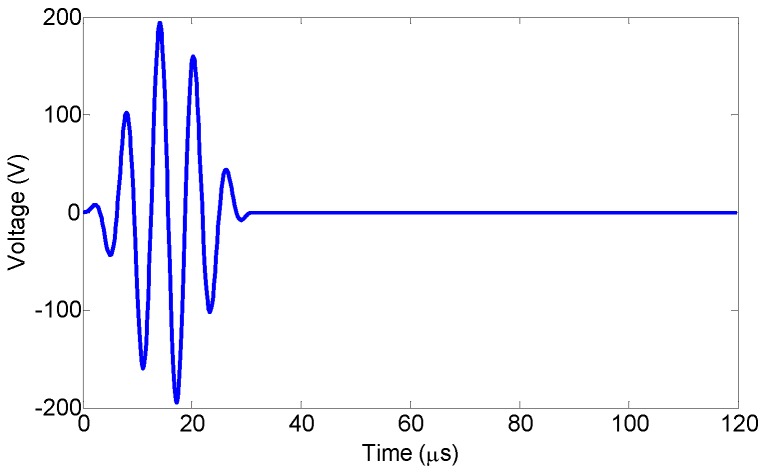
The voltage signal for Lamb waves excitation.

**Figure 6:**
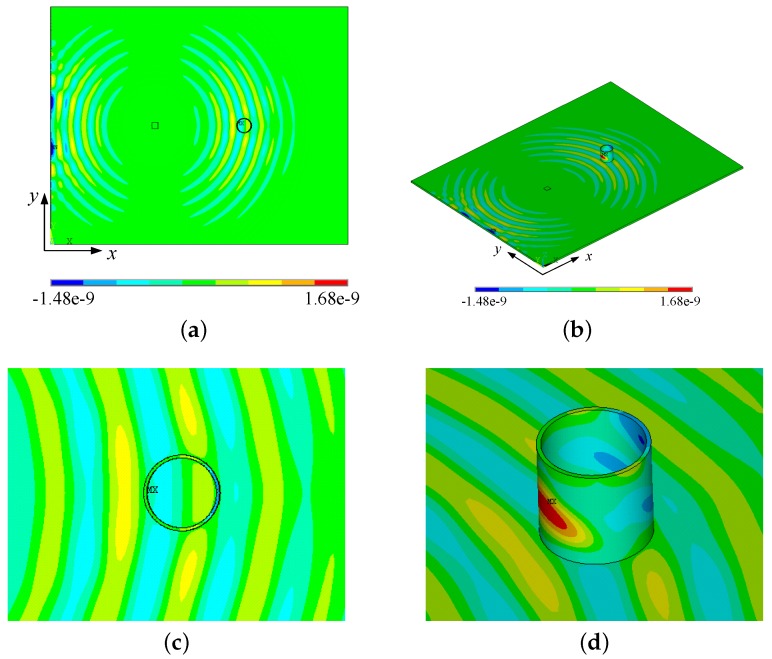
Propagation and scattering of Lamb waves in the aluminum tube: (**a**) top view; (**b**) axonometric view; (**c**,**d**) the corresponding enlarged views.

The narrow-band excitation signal was a 5-cycle sinusoid tone burst, defined by Equation (6), with a fixed center frequency of 160 kHz, as shown in Figure 5.

(6) $$V\left(t\right)=A\left[H\left(t\right)-H\left(t-\frac{N_{cycle}}{f_{c}}\right)\right]\left[1-\cos\left(\frac{2\pi f_{c}t}{N_{cycle}}\right)\right]\sin\left(2\pi f_{c}t\right),$$

where *A* is the amplitude, H(t) Heaviside step function, $f_{c}$ the center frequency of the wave, and $N_{cycle}$ is the number of the signal cycles, and $N_{cycle}=5$ in this work. With two piezoelectric actuators applying an anti-symmetric electric field, only the omnidirectional $A_{0}$ mode of a Lamb wave was generated. This can reduce complexity of the guided waves propagating in the plate. Figure 6 shows the in-plane displacement component *u* contour on the surfaces of the plate and the round tube at the time 40 μs. The guided waves scattering from the plate through the strong coupling between them and propagating on the round tube are clearly visible in Figure 6b. Behind the sensor, most of the energy continues to propagate towards the plate edge.

**Figure 7:**
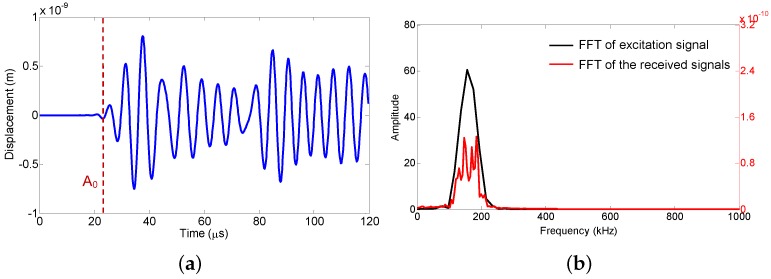
Lamb wave signals and their frequency spectra: (**a**) the displacement component *w*; (**b**) the frequency spectra of the excitation signal and the displacement component *w*, respectively.

For the $A_{0}$ mode of a Lamb wave, the out-of-plane displacement component *w* in the *z*-direction is dominant, so the values of displacement components *w* on the surface of the small tube are extracted and plotted in Figure 7a. The arrival time of the guided wave signal fits well with the value calculated according to the theoretical value, which is shown as a dotted red line in Figure 7a. Owing to the multiple reflections and mode conversions of the ultrasonic waves in the small round tube, after the first major wave packet, the other wave packets cannot be recognized individually. Thus, the single wave packet of a Lamb wave is hardly recognized. From this point of view, the signals received by this UFOS do not reflect the actual shape of the ultrasonic stress waves. However, according to Equation (5), the superposition of multiple packets does not change the frequency characteristic of signals. Figure 7b shows the frequency spectra of the excitation signal and the signal received by UFOS. It can be observed that the signal received by the UFOS can be still considered as the narrow-band, and the main frequency components are contained within the frequency spectrum of the excitation signal. All results obtained from finite element analysis indicate the mechanism and feasibility of the proposed sensor.

## 4. Experimental Investigations on the Specimens

In the following experimental investigations, the proposed UFOS was used to detect Lamb waves propagating in an aluminum plate and receive acoustic emission signals produced in a reinforced concrete beam. The former applications are often found in guided wave based structural health monitoring methods, whereas the latter applications are often found in acoustic emission non-destructive evaluation techniques. In both groups of experiments, the ultrasonic signals were detected by a Mach–Zehnder interferometer, which consisted of a RIO ORION laser module (RIO0175-5-01-3) (Redfern Integrated Optics Inc., Santa Clara, CA, USA), which is a modulation laser source, and a Thorlabs FPD510 photodetector (MenloSystems GmbH, Martinsried, Bavaria, Germany), which converts light signals into electric signals. The output voltage signals from the photodetector were collected at a 2 GHz sampling rate using the digital oscilloscope Tektronix DPO 2024 (Tektronix, Beaverton, OR, USA).

### 4.1. Experiments on the Aluminum Plate

**Figure 8:**
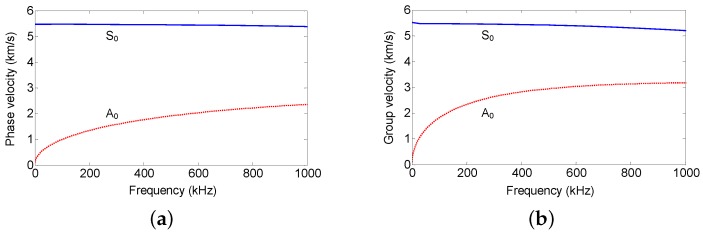
The dispersion curves for the aluminum plate: (**a**) phase velocity vs. frequency; (**b**) group velocity vs. frequency.

**Figure 9:**
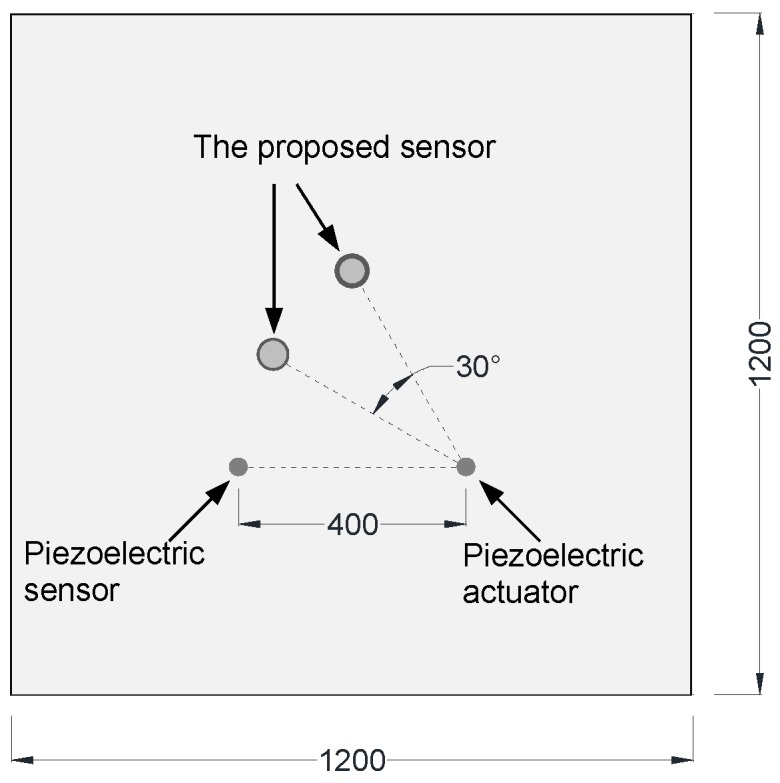
Layout of the actuator and sensors on the aluminum plate in the experimental investigations (unit: mm).

**Figure 10:**
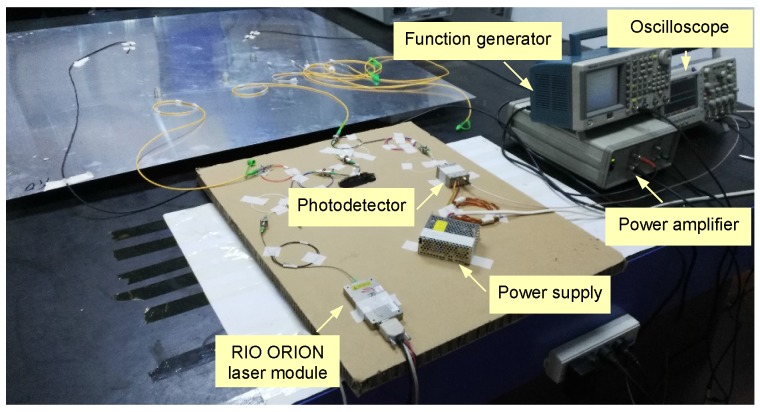
Experimental setup for Lamb waves generation and reception with UFOSs and the piezoelectric wafers.

The aluminum plate is a representative waveguide frequently used in Lamb wave related experiments. In this work, the proposed sensors were also demonstrated on an aluminum plate with dimensions of 1200 mm × 1200 mm × 1 mm. The Lamb wave was excited by a piezoelectric wafer, which was driven by a function generator Tektronix AFG3252 (Tektronix, Beaverton, OR, USA) and a power amplifier TEGAM 2350 (TEGAM, Geneva, OH, USA), and received by two UFOSs and a piezoelectric wafer for the purpose of comparison. Figure 8 shows the dispersion curves of Lamb wave in the aluminum plate. The diameter and thickness of the piezoelectric wafers are 10 mm and 1 mm, respectively. They were connected with the digital oscilloscope with 2 GHz sampling rate. The different dimensions of the two UFOSs are listed in Table 1. The optical fiber wrapped around the tube with superglue is single-mode and the length is approximately 1.88 m (30 loops). The layout and location are shown in Figure 9. The distance between the actuator and sensors is approximately 40 cm. All of the actuator and sensors were attached onto the surface of the plate by superglue, which can be considered as strong coupling. The experimental setup was shown in Figure 10.

**Figure 11:**
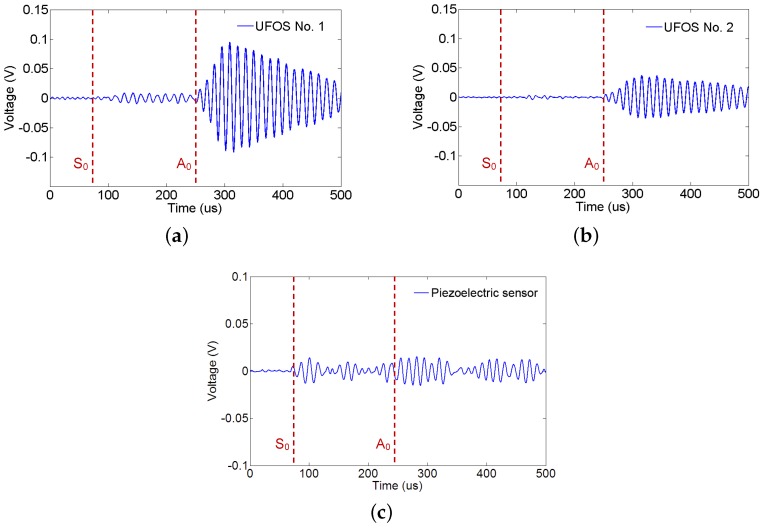
Lamb wave signals from different UFOSs and piezoelectric wafer at 70 kHz for: (**a**) UFOS No. 1; (**b**) UFOS No. 2; (**c**) the piezoelectric sensor.

**Figure 12:**
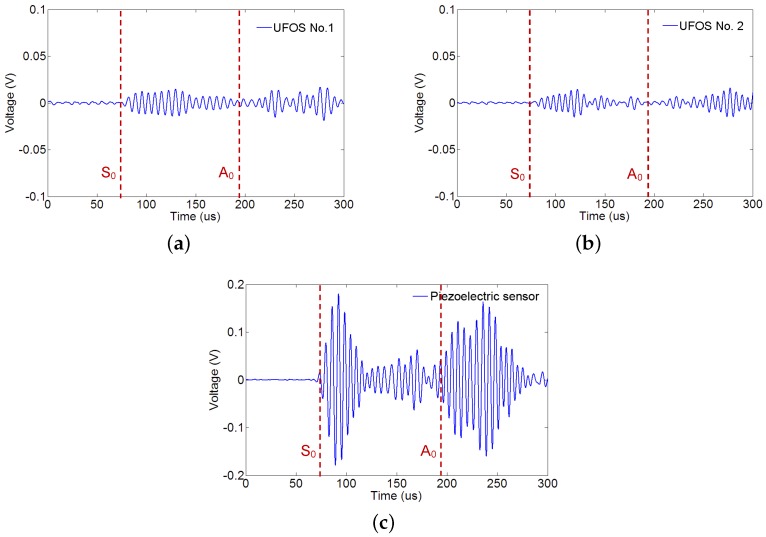
Lamb wave signals from different UFOSs and piezoelectric wafer at 140 kHz for: (**a**) UFOS No. 1; (**b**) UFOS No. 2; (**c**) the piezoelectric sensor.

The Lamb waves were generated from a low frequency of 30 kHz to a high frequency of 240 kHz in increments of 10 kHz, in order to determine the sensitivities to different frequencies. Figure 11 and Figure 12 show the signals received by all the sensors at 70 kHz and 140 kHz, respectively. In all figures, the arrival time of $S_{0}$ and $A_{0}$ modes is marked by the dotted red lines according to the group velocity shown in Figure 8. In Figure 11, the first arrival wave packet is $S_{0}$ mode Lamb wave for both UFOSs and the piezoelectric sensor. In the latter, $S_{0}$ and $A_{0}$ modes may be distinguished, whereas, in the former, these two modes are overlapping owing to the multiple reflections within the sensor body. Nevertheless, if there is a sufficient time interval, these two modes can still be separated until $S_{0}$ mode signal attenuates. The large response of UFOS after 260 μs results from the reflections on all the edges, since their arrival times are similar.

**Figure 13:**
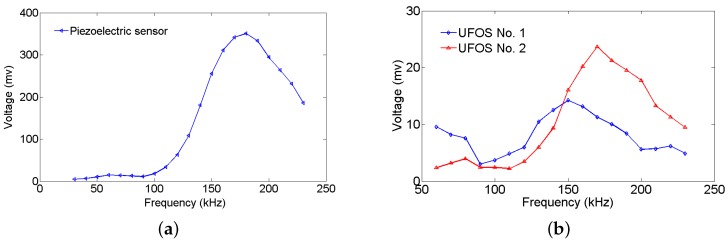
Tuning frequency curves for: (**a**) the piezoelectric sensor; (**b**) UFOSs No.1 and No. 2.

In Figure 12, which is the higher frequency case, the piezoelectric sensor exhibits a larger response, which is also observed in Figure 13. The maximum values of the first wave packet in both the piezoelectric sensor and UFOSs are selected and plotted in this figure. These are the “tuning frequency curves” (for $S_{0}$ mode only in this case), which demonstrate that the piezoelectric sensor has a lower sensitivity at low frequency, whereas the UFOSs exhibit high sensitivity at both low and high frequencies. Specifically, the UFOS with a thinner wall exhibits higher sensitivity in the low frequency range, but lower sensitivity in the high frequency range, whereas the opposite is true for the UFOS with a thicker wall. This can be attributed to the smaller structural stiffness of the UFOS with a thinner wall, which results in a lower resonant frequency and a larger response. Finally, although the sensitivity of the UFOS is lower than that of the piezoelectric sensor, it can be increased further by using a longer optical fiber. Moreover, the tuning frequency curve of the proposed sensor is related to the mode coupling effects, interferometer drift, etc.

### 4.2. Experiments on the Reinforced Concrete Beam

**Figure 14:**
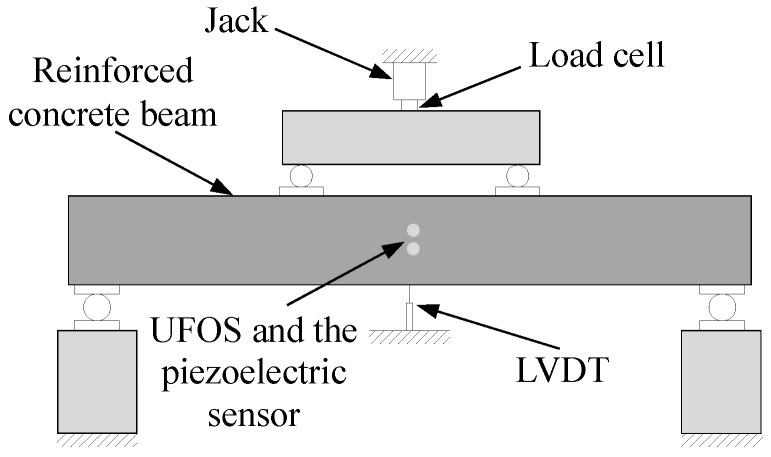
Experimental setup for acoustic emission tests on the reinforced concrete beam.

Reinforced concrete is the most common civil engineering material. The acoustic emission technique is one of the most effective methods for the crack monitoring of reinforced concrete structures [41,42,43]. In this work, a reinforced concrete beam with width 150 mm, depth 250 mm, and length 2000 mm was cast. The beam contained three tensile reinforcing bars with a diameter of 14 mm. The tested beams were instrumented with one UFOS and one piezoelectric sensor placed at approximately the mid span for the purpose of comparison. Figure 14 shows the sketch of experimental setup. The DiSP-4/PCI system, a product of the Physical Acoustic Corporation (Princeton Junction, NJ, USA), was used in this work to collect the signals from the piezoelectric acoustic emission sensor. This sensor is model R15α. The preamplifier used in the DiSP-4/PCI system has a gain of 40 dB. Moreover, the sampling rate for the piezoelectric sensor is 5 MHz. In the following tests, the UFOS with a wall and base thickness of 0.5 mm was used. The Mach–Zehnder interferometer used in this test is the same as the above. Acoustic emission signals were produced via two methods: striking the beam with a steel bar first and subsequently producing cracks by static loading.

**Figure 15:**
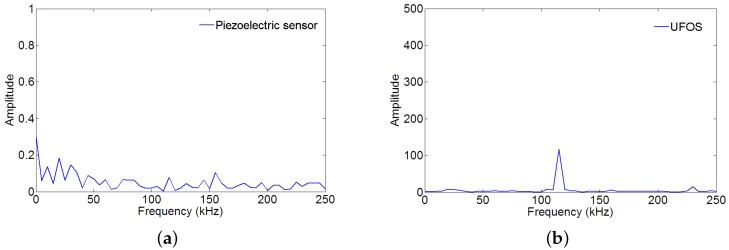
The frequency spectra of noise during the acoustic emission tests for: (**a**) the piezoelectric sensor; (**b**) UFOS.

Since the noise affected the signals in both time and frequency domains, the noise signals were collected by a data acquisition system and their frequency spectra were analyzed before the tests, such that they can be distinguished from the acoustic emission signals. As shown in Figure 15, the noise in the piezoelectric sensor is very faint, whereas, in the UFOS, there is high noise at the frequency of approximately 114 kHz, which appeared in the frequency spectrum of the piezoelectric sensor signal. The noise was not isolated from the acoustic emission signals in the following tests, but this figure provides the useful information for understanding the following signals.

**Figure 16:**
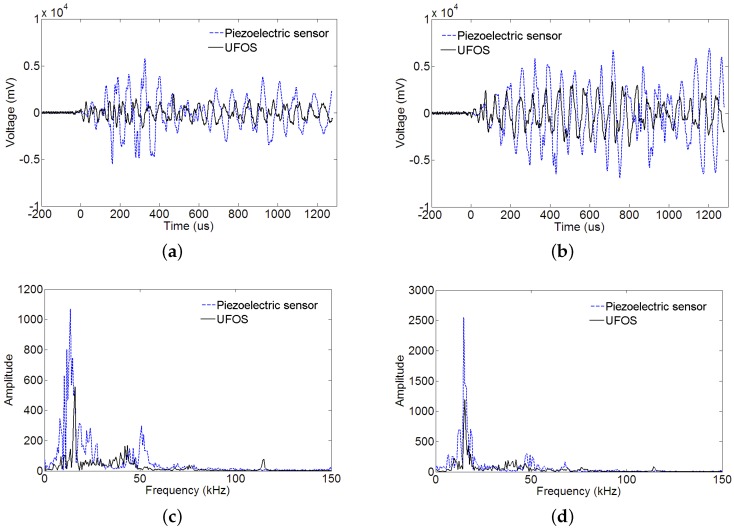
Acoustic emission signals produced by striking the reinforced concrete beam: (**a**,**b**) the acoustic emission signals for two times; (**c**,**d**) the corresponding Fourier spectra.

**Figure 17:**
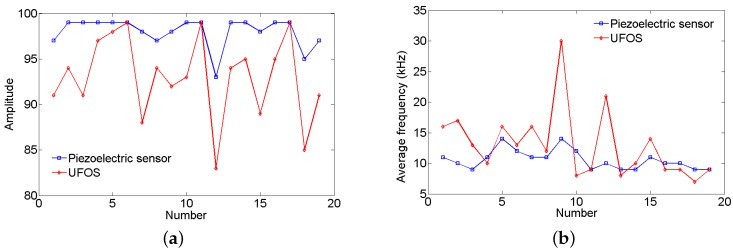
The characteristics of acoustic emission signals for both sensors during striking the beam: (**a**) amplitude vs. number; (**b**) average frequency vs. number.

The surface of the reinforced concrete beam was struck using a steel bar for two times, and the acoustic emission signals were collected and shown in Figure 16a,b. The proposed UFOS detected acoustic emission signals successfully, but the amplitudes are a little less than those of the piezoelectric acoustic emission sensor. Since the signals in the time domain are hard to compare, the frequency spectra are given in Figure 16c,d for both signals. From the curves in the frequency domain, it can be observed that the UFOS has similar frequency components as the piezoelectric sensor in the low frequency range, but slightly more components in the high frequency range. Figure 17 further shows that the acoustic emission signals detected by the UFOS have lower amplitude but higher average frequency. In this figure, the *x*-axis denotes the number of acoustic emission events.

**Figure 18:**
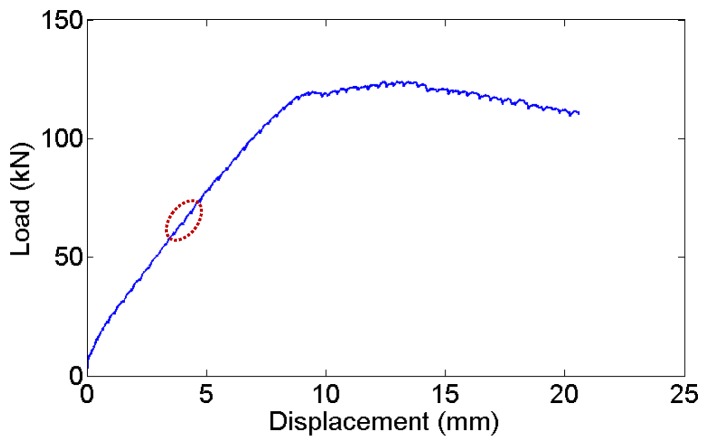
Load–displacement behavior of the reinforced concrete beam.

**Figure 19:**
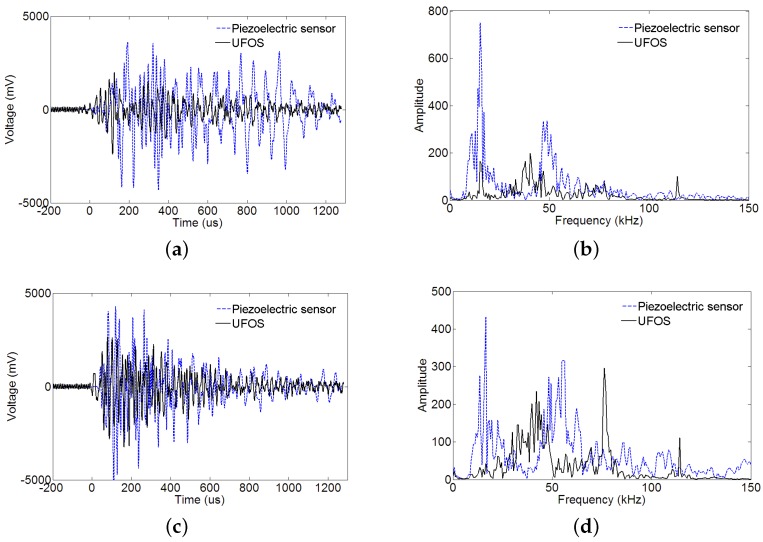
Acoustic emission signals produced by cracks extension: (**a**,**b**) the acoustic emission signals and their Fourier spectra for crack 1; (**c**,**d**) the acoustic emission signals and their Fourier spectra for crack 2.

**Figure 20:**
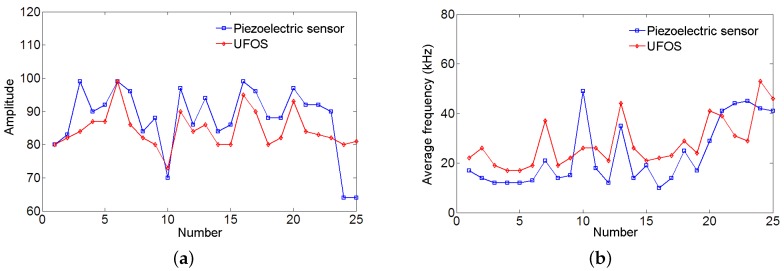
The characteristics of acoustic emission signals for both sensors due to cracks’ extension: (**a**) amplitude vs. number; (**b**) average frequency vs. number.

Furthermore, the reinforced concrete beam was tested under four-point loading. The loading was applied manually by a 320 kN jack and the displacement speed was relatively constant, as shown in Figure 18. The load was recorded by a load cell, and the displacement in the middle section of the beam was measured using a linear variable differential transformer (LVDT). The load versus displacement curve is illustrated in Figure 18. The following figures (Figure 19) show two groups of acoustic emission signals, which are obtained during the failure process of the reinforced concrete beam. The rough area, where the signals in Figure 19 were obtained, is shown as a red dotted oval in Figure 18. The other frequency components show that both sensors can catch the signal emitting from the tensile cracks below approximately 100 kHz, which is the typical frequency range of the tensile cracks of concrete materials. While the detailed frequency is different between the UFOS and piezoelectric sensor, this can be attributed to the differences in their detailed sensitivity curves. Figure 20 demonstrates that the characteristics of acoustic emission signals from both sensors are similar, as shown previously.

It is known that piezoelectric acoustic emission sensors cannot measure the accurate structural deformation, such as the structural strain, according to their working principles. Similarly, for the UFOS proposed in this work, the voltage amplitudes of signals do not have a simple relationship with the structural strain or deformation, but the signals collected by them can be used to evaluate the structural damage certainly. Thus, the proposed sensor is more suitable to detect acoustic emission events.

## 5. Conclusions

In this paper, a 3D shaped UFOS is proposed to sense ultrasonic stress waves, including guided waves in the active damage detection methods and acoustic emission signals, which are collected usually in the passive damage detection methods. Based on the principle of a Mach–Zehnder interferometer, the UFOS is obtained by wrapping an optical fiber on a hollow cylinder with a base. By being attached onto the surfaces of structures, the UFOS can catch the wave signals through strong coupling and wave scattering.

First, an analytical model was developed for the proposed UFOS. The sensitivity of the 3D UFOS also depends on its sensing length, and it can be realized with relatively limited space and long optical fiber (up to several meters). Subsequently, its feasibility for the detection of guided ultrasonic waves was demonstrated using finite element analyses. The results showed that the frequency characteristics of the detected signals were affected slightly, as indicated by the formula. Experiments conducted on the plate indicated that the UFOSs used in this work exhibit smaller responses than the piezoelectric sensor, but they can be improved by increasing the optical fiber length. Moreover, the UFOS with a thinner wall has higher sensitivity in the low frequency range as compared to the UFOS with a thicker wall. Experiments conducted on the reinforced concrete beam further indicate that the UFOS can detect the acoustic emission signals with a reasonable arrival time and frequency range in comparison with the results from the piezoelectric sensor.

## Figures and Tables

**Table 1:** Dimensions of UFOSs (unit: mm).

Sensor No.	Outside Diameter	Height	Wall Thickness	Base Thickness
1	20	20	0.5	1.0
2		20	1.0	1.0

## Abbreviations

The following abbreviations are used in this manuscript:

AE	Acoustic emission
EMAT	Electromagnetic acoustic transducer
FOS	Fiber optic sensor
UFOS	Ultrasonic fiber optic sensor
LVDT	Linear variable differential transformer
